# Is There Any Relationship between Vitamin D Deficiency and Gross Motor Development in 12-Month-Old Children?

**Published:** 2019

**Authors:** Reza TAVAKOLIZADEH, Maryam ARDALANI, Ghazal SHARIATPANAHI, Sayed Yousef MOJTAHEDI, Azadeh SAYARIFARD

**Affiliations:** 1 Ziaeeyan Hospital, Tehran University of Medical Sciences, Tehran, Iran; 2 Bahrami Children Hospital, Tehran University of Medical Sciences, Tehran, Iran; 3 Growth and Development Research Center, Tehran University of Medical Sciences, Tehran, Iran

**Keywords:** Vitamin D, Gross motor development, Children

## Abstract

**Objective:**

We aimed to assess the possible relationship between vitamin D deficiency and gross motor developmental milestones in Iranian children in Tehran City, Iran.

**Materials & Methods:**

In this cross-sectional study, 186 healthy children of one-yr-old age referring to a children hospital in Tehran, Iran from May 2015 to April 2016, were enrolled. The gross motor developmental milestone of children and their serum vitamin D concentration were evaluated.

**Results:**

Overall, 186 children, 92 males (49.5%) were studied. Twenty-four patients (12.9%) just were able to sit without support, and 40 patients (21.5%) to stand alone and to sit without support. Besides, 122 patients (65.6%) were able to walk with assistant or alone and to sit without support and stand alone. Vitamin D was sufficient in 148 children (79.6%), insufficient in 32 (17.2%) and deficient in 6 (3.2%). Sufficient vitamin D was significantly correlated with walking ability (*P*<0.001, OR=3.9, 95%CI=1.9-8.4).

**Conclusion:**

Considering the significant correlation between vitamin D deficiency and gross motor developmental milestones in this population of children, children referring to gross motor developmental delay need to be thoroughly evaluated for vitamin D deficiency.

## Introduction

Vitamin D is important for skeletal growth and cellular function due to its role in calcium homeostasis through regulating calcium absorption in bowel (1). Additional health benefits of vitamin D have been anticipated in children such as prevention of type 1 diabetes mellitus, asthma, cardiovascular disease, influenza and respiratory infections (-). In children, vitamin D has a role in motor development (6, 7).

Vitamin D deficiency is a common condition in children. Particularly those who have chronic disease, malnourished, have geographically limited sun exposure, as well as those with darker skin, and chronically using special medications(8, 9). 

In case of vitamin D deficiency, bowel absorption of calcium and phosphorus is reduced and the parathyroid gland responds to low serum calcium levels by producing parathyroid hormone (PTH) to increase the serum calcium to an acceptable level. PTH increases calcium reabsorption in the kidneys and excretion of phosphorus, thus reducing the adverse complication of elevated calcium phosphate products (10, 12). Over weeks to months stunting, rickets and osteomalacia might appear (13, 14). Children`s vitamin D insufficiency also may prevent children achieving their peak bone mass and genetically programmed height (13). The enhanced rate of bone growth in children implies how inadequate level of vitamin D in this period of life can damage this process (8). 

Vitamin D status is important for motor development in children (6, 7); however, more researches are needed to evaluate the association of vitamin D status in pediatric patients with developmental status (15).

The prevalence of vitamin D deficiency is reported considerably higher in Middle-Eastern countries. The incidence of Vitamin D deficiency among Iranian children is 85.6% (16, 17).

Even though there is high prevalence of Vitamin D deficiency among Iranian children; based on our knowledge the relation between Vitamin D levels and motor development in children has not ever been evaluated in Iran. Therefore, in this study, we aimed to assess the possible relationship between vitamin D deficiency and gross motor developmental milestones in one-year-old Iranian children in Tehran City.

## Materials & Methods

In this cross-sectional study, 186 twelve-month-old children referred to a university hospital in Tehran, Iran from May 2015 to April 2016, were enrolled. 

The study was approved by the Research Ethics Committee of Tehran University of Medical Sciences, Tehran, Iran. Informed parental consent was obtained prior to sampling. 

The inclusion criteria included term delivery, twelve-month-old, without any history of asphyxia, neuromuscular disease, metabolic diseases, Down syndrome and any previous major disease.

The sampling was convenience. Demographic data such as gender were recorded. Gross motor development was assessed by a pediatrician.

Three distinct gross motor milestones were selected as follows: sitting without support; standing alone and walking with assistant or alone. These three milestones were selected considering that most of twelve-month-old children have the ability to perform them based on various existing developmental scale (18, 19). Moreover, they are universal and simple to test and evaluate. Therefore, administration and interpretation of the child’s performance could be cleared (18, 19).

**Table 1:** The relation between gender and serum levels of vitamin D with gross motor development

		Gross motor development			*P*-value
		Sitting without support (%)	Standing alone (%)	Walking with assistant or alone (%)	
Gender	Male	57 (46.7)	22 (55)	13 (54.2)	0.58
	Female	65 (53.3)	18 (45)	11 (45.8)	
Vitamin D status	Deficiency	1 (0.8)	1 (2.5)	4 (16.7)	<0.001
	Insufficiency	14 (11.5)	10 (25)	8 (33.3)	
	Sufficient	107 (87.7)	29 (72.5)	12 (50)	

**Table 2:** The prediction value of gender and serum level of vitamin D to the gross motor development

Gross Motor Development^a^		B	OR	*P*-value	95% CI for OR
Walking with assistant or alone	Intercept	-1.83		0.17	
	Gender	0.29	1.34	0.53	0.52 – 3.45
	Vitamin D sufficient	3.58	35.95	0.002^*^	3.69 – 349.87
	Vitamin D insufficient	1.96	7.1	0.1	0.67 – 75.39
	Vitamin D deficit	0^b^	.	.	.
Standing alone	Intercept	-1.34		0.32	
	Gender	-0.02	0.97	0.96	0.34 – 2.78
	Vitamin D sufficient	2.26	9.66	0.052	0.97 – 95.62
	Vitamin D insufficient	1.6	4.99	0.18	0.46 - 54
	Vitamin D deficit	0^b^	.	.	.

B: Coefficient

OR: Odds ratio

CI: Confidence Interval

a : The reference category is sitting without support

b : this parameter is set to zero because it is redundant.

**Vitamin D assessment:** Serum level of Vitamin D was measured by ELISA method (using Bioactiva Diagnostica kits). Then, children were divided into 3 groups based on serum level of Vitamin D:

- Deficient: 25(OH)D < 20 ng/ml
- Insufficient: 21 ng/ml < 25(OH)D <29 ng/ml
- Sufficient: > 30 ng/ml (20).

**Data analysis**

Statistical analyses were carried out using SPSS 18 (Chicago, IL, USA). The frequency of qualitative variables was reported. For evaluation of relationships between qualitative variables, Chi-squared test was used. In all tests, *P*-value<0.05 was considered significant difference. Finally, to predict the effect of gender and vitamin D status on the gross motor developmental milestones in one-year-old children, multinomial logistic regression test was applied. 

## Results

Overall, 186 children of one-yr-old age, including 92 boys (49.5%) and 94 girls (50.5%) were studied. Twenty-four patients (12.9%) just were able to sit without support, 40 patients (21.5%) were able to stand alone, and to sit without support. Besides, 122 patients (65.6%) were able to walk with assistant or alone, in addition to sit without support and stand alone.

Vitamin D was sufficient in 148 children (79.6%), insufficient in 32 (17.2%) and deficient in 6 (3.2%). There was no significant relationship between gender and gross motor development (*P*=0.58). There was a significant relation between Vitamin D status and motor development in our participants (*P*<0.001). Of six children with Vitamin D deficiency, one case (16.7%) could walk and 1 case (16.7%) could stand alone. Four cases (66.7%) were able to sit without support (Table 1).

Table 2 shows the result of multinomial logistic regression test. Gender did not have a significant effect to predict the gross motor developmental milestones in one-year-old children. The probability of walking for a child who had sufficient Vitamin D was 35 times more than a child who had Vitamin D deficiency (*P*=0.002, OR=35.95, 95% CI=3.69-349.87).

## Discussion

One common clinical symptom in Vitamin D-associated rickets is motor developmental delay in children (6, 7). 

In the present study, 12.9% of children were able to sit without support, 21.5% were able to stand alone and 65.6% were able to walk. In case of serum levels of Vitamin D, only 6 participants had Vitamin D deficiency (3.2%). There was a significant relation between Vitamin D status and gross motor development in this population of children (*P*<0.001). 

According to the results of Multinomial Logistic Regression analysis, the chance of walking ability in children with Vitamin D sufficient is up to 35 folds higher than children with Vitamin D deficiency (*P*=0.002). 

A retrospective study on children suffering from hypocalcemia or rickets because of vitamin D deficiency showed that Vitamin D deficiency could play a role in manifestations such as leg parenthesis, joint stretching, abnormal bones and motor developmental delay (21).

In another study in children with rickets, the clinical manifestations were motor developmental delay in 20 cases (33.3%), skeletal changes in clinical examination (66.66%) in 40 infants and radiologic signs of rickets in 51 cases (85%) were confirmed (22). 

Overall, 24 under five years old children suffering from Vitamin D deficiency and motor developmental delay were the most important clinical signs among children with Vitamin D deficiency (23).

Totally, 42 infants older than 1 year were evaluated, who were not able to walk. About 60% of these children had nutritional rickets, and all of them showed normal radiologic and laboratory findings after 3 wk of treatment. Seventeen patients were able to walk 3 months after treatment (6). Moreover, walking delay was reported as one of severe Vitamin D deficiency complications (24). In England 17 children hospitalized because of secondary hypocalcemic convulsion due to Vitamin D deficiency were studied and showed delay in motor development especially walking as the most common complication (25). Vitamin D deficiency in children can be the cause of gross motor delay among them and both of them were consistent. 

**In conclusion, **Vitamin D deficiency in children is associated with motor development delay. Children referring to physicians with motor developmental delay need to be checked for Vitamin D, in order to administer appropriate doses of Vitamin D and prevent further continuation of motor developmental delay.

## References

[1] Fleet JC, Schoch RD (2010). Molecular mechanisms for regulation of intestinal calcium absorption by vitamin D and other factors. Crit Rev Clin Lab Sci.

[2] Braegger C, Campoy C, Colomb V, Decsi T, Domellof M, Fewtrell M (2013). Vitamin D in the healthy European paediatric population. J Pediatr Gastroenterol Nutr.

[3] Christakos S, DeLuca HF (2011). Minireview: vitamin D: is there a role in extraskeletal health?. Endocrinology.

[4] Bouillon R, Norman AW, Lips P (2007). Vitamin D deficiency. N Engl J Med.

[5] Wang TJ, Pencina MJ, Booth SL, Jacques PF, Ingelsson E, Lanier K (2008). Vitamin D deficiency and risk of cardiovascular disease. Circulation.

[6] Agarwal A, Gulati D, Rath S, Walia M (2009). Rickets: a cause of delayed walking in toddlers. Indian J Pediatr.

[7] Soliman A, De Sanctis V, Adel A, El Awwa A, Bedair S (2012). Clinical, biochemical and radiological manifestations of severe vitamin d deficiency in adolescents versus children: response to therapy. Georgian Med News.

[8] Lee JY, So T-Y, Thackray J (2013). A review on vitamin d deficiency treatment in pediatric patients. J Pediatr Pharmacol Ther.

[9] Misra M, Pacaud D, Petryk A, Collett-Solberg PF, Kappy M (2008). Vitamin D deficiency in children and its management: review of current knowledge and recommendations. Pediatrics.

[10] Moe SM (2008). Disorders involving calcium, phosphorus, and magnesium. Prim Care.

[11] Trivedi DP, Doll R, Khaw KT (2003). Effect of four monthly oral vitamin D3 (cholecalciferol) supplementation on fractures and mortality in men and women living in the community: randomised double blind controlled trial. BMJ.

[12] Holick MF, Binkley NC, Bischoff-Ferrari HA, Gordon CM, Hanley DA, Heaney RP (2011). Evaluation, treatment, and prevention of vitamin D deficiency: an Endocrine Society clinical practice guideline. J Clin Endocrinol Metab.

[13] Holick MF (2007). Vitamin D deficiency. N Engl J Med.

[14] Zhou C, Assem M, Tay JC, Watkins PB, Blumberg B, Schuetz EG (2006). Steroid and xenobiotic receptor and vitamin D receptor crosstalk mediates CYP24 expression and drug-induced osteomalacia. J Clin Invest.

[15] Filteau S, Rehman AM, Yousafzai A, Chugh R, Kaur M, Sachdev HP (2016). Associations of vitamin D status, bone health and anthropometry, with gross motor development and performance of school-aged Indian children who were born at term with low birth weight. BMJ Open.

[16] Motlaghzadeh Y, Sayarifard F, Allahverdi B, Rabbani A, Setoodeh A, Sayarifard A (2016). Assessment of Vitamin D Status and Response to Vitamin D3 in Obese and Non-Obese Iranian Children. J Trop Pediatr.

[17] Mohammadian S, Mortezazadeh R, Zaeri H, Vakili MA (2014). Relationship between 25-hydroxy vitamin-D and obesity in 2-7 years old children referred to a paediatric hospital in Iran. Journal of clinical and diagnostic research: JCDR.

[18] Wijnhoven TMA, Onis Md, Onyango AW, Wang T, Bjoerneboe G-EA, Bhandari N (2004). Assessment of Gross Motor Development in the WHO Multicentre Growth Reference Study. Food Nutr Bull.

[19] WHO Multicentre Growth Reference Study Group WHO Motor Development Study: windows of achievement for six gross motor development milestones. Acta Paediatr Suppl 2006 Apr.

[20] Marcdante K, Kliegman RM, Behrman RE, Jenson HB (2010). Nelson Essentials of Pediatrics.

[21] Ladhani S, Srinivasan L, Buchanan C, Allgrove J (2004). Presentation of vitamin D deficiency. Arch Dis Child.

[22] Siddiqui TS, Rai MI (2005). Presentation and predisposing factors of nutritional rickets in children of Hazara Division. J Ayub Med Coll Abbottabad.

[23] Callaghan AL, Moy R, Booth IW, Debelle G, Shaw NJ (2006). Incidence of symptomatic vitamin D deficiency. Arch Dis Child.

[24] Soliman AT, El-Dabbagh M, Adel A, Al Ali M, Bedair EMA, ElAlaily RK Clinical responses to a mega-dose of vitamin D3 in infants and toddlers with vitamin D deficiency rickets. J Trop Pediatr 2010 Feb.

[25] Michie C, Bangalore S (2010). Managing vitamin D deficiency in children. London J Prim Care (Abingdon).

